# The first transcriptome of Italian wall lizard, a new tool to infer about the Island Syndrome

**DOI:** 10.1371/journal.pone.0185227

**Published:** 2017-09-27

**Authors:** Martina Trapanese, Maria Buglione, Valeria Maselli, Simona Petrelli, Serena Aceto, Domenico Fulgione

**Affiliations:** Department of Biology, University of Naples Federico II, campus of Monte Sant’Angelo, Naples, Italy; Xiamen University, CHINA

## Abstract

Some insular lizards show a high degree of differentiation from their conspecific mainland populations, like Licosa island lizards, which are described as affected by Reversed Island Syndrome (RIS). In previous works, we demonstrated that some traits of RIS, as melanization, depend on a differential expression of gene encoding melanocortin receptors. To better understand the basis of syndrome, and providing raw data for future investigations, we generate the first *de novo* transcriptome of the Italian wall lizard. Comparing mainland and island transcriptomes, we link differences in life-traits to differential gene expression. Our results, taking together testis and brain sequences, generated 275,310 and 269,885 transcripts, 18,434 and 21,606 proteins in Gene Ontology annotation, for mainland and island respectively. Variant calling analysis identified about the same number of SNPs in island and mainland population. Instead, through a differential gene expression analysis we found some putative genes involved in syndrome more expressed in insular samples like *Major Histocompatibility Complex class I*, *Immunoglobulins*, *Melanocortin 4 receptor*, *Neuropeptide Y* and *Proliferating Cell Nuclear Antigen*.

## Introduction

Reptile studies on diverse and complex traits are extremely useful for understanding the modes and timing of evolution. Moreover, reptiles, and lizards in particular, show a high plasticity of phenotypic modulation in response to external selection pressures, such as thermal changes, food stress or unpredictable environments [1–4].

Some lizards on islands show extraordinary diversification from mainland populations and can in fact chart the course of evolution over short time periods, directly measuring the extent to which natural selection changes in strength and direction over time [5]. Such extraordinary plasticity reveals itself in a diverse collection of adaptations in isolated populations that have been widely described for the Antille [6–11], Baleari [12,13], Tyrrhenian and Ionian islands [14–17].

**Fig 1:**
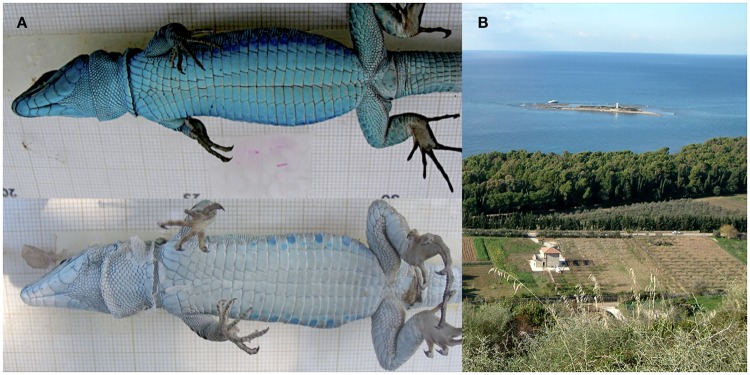
Italian wall lizard color pattern and study area. (A) Lizards from Licosa Island (on top) and from Mainland (on the bottom). (B) Licosa island and its facing mainland (Mediterranean Sea).

The Italian wall lizard (*Podarcis siculus* also known in the literature as *Podarcis sicula*) ranges throughout Italy to the south of the Alps, including Sicily, Sardinia, and many other islands in the Tyrrhenian Sea [18]. Some populations living on islets exhibit a suite of traits involving melanization, which have been described as Reversed Island Syndrome, RIS (Fig 1). According to syndrome, insular lizards show higher food intake rates, aggressiveness, high sexual dimorphism and, in general, invest more resources into reproduction, compared to mainland relatives. RIS affects life history trade-off as a consequence of differential selection rather than genetic drift [14,15,19] as revealed by differential expression of some melanocortin receptor genes [15,20].

With this in mind, we hypothesize that adaptations to island life depend on a regulatory solution rather than genomic mutation, summarizing in a different use of the genome by the insular populations. To investigate as well as to provide raw data about genomic make-up in responses to environmental and evolutionary pressures, we used high-throughput sequence analysis to generate *de novo* transcriptome assemblies. The transcriptome was generated for island and mainland lizards using testis and brain tissue in agreement with the main phenotypic traits encountered in the syndrome concerning to behavior and reproduction.

The *de novo* assembly of a transcriptome is a critical step, in particular when working with high-throughput sequence data in species for which a reference genome is not available, as in this case. If these data are assembled accurately and efficiently, they can be useful for developing markers for understanding population structure, and more generally, for identifying genes and mutations involved in reptile evolution.

## Materials and methods

### Study area

We focus our study on two previously described related populations of Italian wall lizard in the South of Italy, inhabiting Licosa island (ca. 0.8 ha in surface area, geographical coordinates: 40°15′04.23"N, 14°54′01.64"E), and its facing mainland (Punta Licosa, 40°15′06.15"N, 14°54′19.68"E).

### Sampling

We analyzed transcriptome of two lizard’s ecotypes in triplicate (3 lizards from mainland and 3 lizards from island) using adult males of comparable age. Lizards were aged using their snout-vent length according to the growth rate defined by skeletochronology (Additional file in [15]). The animals were kept according to the authorization by the Ministry of the Environment and Protection of Land and Sea (also known as MATTM) (prot. 4363/2015). This authorization was subsequently recognised by the Cilento National Park. Lizards were collected by nylon loop. To minimize the demographic impact we worked just on individuals which were dead during capture and manipulation. Then they were immediately cryopreserved in liquid nitrogen. Experimental procedures were approved by the Ethical Committee for Animal Experiments, University of Naples Federico II (ID: 2013/0096988), and according to Italian law.

### RNA isolation

Total RNA was isolated from tissues (brain and testis of each lizard) using TRI Reagent (EuroClone, Milan, Italy) according to the manufacturer’s instructions. The RNA quality and quantity were determined using Agilent Bioanalyzer 2100 (Agilent Technologies, Santa Clara, CA, USA) and Nanodrop spectrophotometer (Thermo Scientific Inc., Waltham, MA, USA) respectively.

### Library preparation and sequencing

The sequencing, including sample quality control, was performed by Genomix4life S.R.L. (Baronissi, Salerno, Italy). Indexed libraries (using index-tagged samples) were prepared from 1 ug of each purified RNA sample using TruSeq Stranded mRNA Sample Prep Kits (Illumina, San Diego, CA, USA) according to the manufacturer’s instructions. A sequence index is useful to tag each sample in unique manner, so after pooling it is possible to identify each of them. Libraries were quantified using an Agilent Bioanalyzer 2100 (Agilent Technologies, Santa Clara, CA, USA) and pooled such that each index-tagged sample was present in equimolar amounts, with a final concentration of pooled samples of 2 nM. The pooled samples were then subjected to cluster generation and sequenced using an Illumina HiSeq 2500 System (Illumina, San Diego, CA, USA) in a 2x100 paired-end format at a final concentration of 8 pmol.

The quality of raw sequencing reads was assessed using FastQC (http://www.bioinformatics.babraham.ac.uk/projects/fastqc/), followed by removal of Illumina adapters and trimming using Trimmomatic (v0.33) [21]. Trimming was performed at both the 5’- and 3’-end of reads, eliminating bases with a Phred score of less than 35. Additionally, the first 10 bases from the 5’-end of all reads were cropped, and only reads with a minimum length of 35 nt were retained. Paired reads were processed simultaneously, and orphan reads were removed. The high quality reads were used as input to perform transcriptome assembly after normalization (with Trinity v2.1.1 [22]). Read normalization reduces the redundancy of the dataset thus speeding the analysis and increasing the quality of the assembly.

### De novo sequence assembly

The sequence assembly was performed using TransABySS (v.1.5.3) [23] and Trinity (v2.1.1) [22], discarding transcripts shorter than 250 bp. Comparisons of short-read assembly programs have shown that these two programs are effective at producing quality assemblies of short-read transcriptome data. Both TransABySS and Trinity were set to perform strand-specific assemblies. Additionally, Trinity was set with the options [—min_kmer_cov 2,—min_per_id_same_path 95 and—max_diffs_same_path 4] and TransABySS with the option [—pid 0.9].

Because transcriptome coverage is highly variable due to the expression levels of the genes, there is no single kmer length that will provide an optimal assembly for a transcriptome [24]. Highly expressed transcripts will assemble better with longer kmer lengths, whereas lowly expressed transcripts will be better assembled if shorter kmer lengths are used. Thus, we ran TransABySS over a range of kmers (i.e., 25, 47 and 71) and merged these assemblies to produce a non-redundant set of contigs with TransABySS-merge. Transcriptome assemblies were performed separately on each sample. Each assembly was then analyzed with Transrate (v1.0.1) [25] together with the reads to calculate assembly statistics and filter possible artifacts. The transcripts from each assembly passing the Transrate filter were then merged using TransABySS-merge to obtain a non-redundant reference transcriptome.

### Assembly filtering and annotation

To remove possible contaminants from the assembled transcriptome, sequences were blasted (v.2.2.30) against the NCBI NT database (minimum e-value set to 0.1 and -max_target_seqs 1). The BLAST results were then filtered, keeping only sequences with an alignment/transcript length ratio higher than 20%. The taxonomic classification of each BLAST hit was obtained, and all transcripts with a best hit whose taxa were not included in the Sauria, were discarded from the assembly.

The putative proteins encoded by the assembled transcripts were identified using TransDecoder (v2.0.1) [26]. First, the LongOrfs tool was used to identify all possible protein sequences of a length greater than 50 aa in strand-specific mode. The recovered sequences were analyzed using BLASTp against the NCBI NR database and HMMScan (v3.1b2) [27] against the PFAM database of the domain of Hidden Markov Models (downloaded on December 2015). The results of both analyses were used as inputs for the TransDecoder Predict tool to obtain the final dataset of proteins.

Gene Ontology (GO) classification was performed using Interproscan (v5) [28] on the predicted protein sequences with PFAM and PANTHER as reference databases. Transcripts containing repetitive elements were identified using RepeatMasker version open-4.0.5 [29]. Repeat-masker was run in default mode with rmblastn version 2.2.25+, and the query species were categorized as “other vertebrate”.

### Variant calling

To study the polymorphisms in island population a variant calling analysis was performed using the available RNA-seq data. The mainland population transcriptome was used as reference, and the reads of each island individual were mapped against the reference, using STAR version 020201 [30], and the resulting BAM files were analyzed with the tool SUPER version 1.0 [31] to identify SNPs. Only variants supported by at least 6 reads and with a quality higher than 30 were kept. A Gene Ontology Enrichment analysis (GOEA) of polymorphic genes was performed using in-house pipelines to identify the gene functions that were most affected by variants. The non-synonymous substitution rates (Ka) and synonymous substitution rates (Ks) were also estimated for orthologous gene pairs between island and mainland populations.

To identify orthologous among the full length and annotated transcripts including SNPs, the Best Reciprocal Hits (BRH) were obtained from pairwise all-versus-all BLASTp search. Later, we calculated Ka and Ks using KaKs Calculator 2.0 tool [32] based on MYN model [33].

### Differential gene expression analysis

From our previous works [14,15] we observed that individuals under syndrome can show greater resistance to stressors, are much more aggressive, sexually hyperactive and have higher food intake rather than their mainland relatives. Therefore, we chose to focus this first analysis on a suite of genes that could be linked to these typical traits, as:

- MHC1, *Major Histocompatibility Complex class I*, is involved in response to cellular stress. It may play a part in innate immunity or in the induction of immune responses [34];
- The Igs (IgD, IgM, IgY), *Immunoglobulins*, are unique characteristic of the adaptive immune systems of all jawed vertebrates, like fishes, amphibian, reptiles, birds and mammals [35,36]. These molecules, in general are responsible for humoral immunity and defend individuals against infections [37–42], but recent studies showed that they have a different role in the plasma membrane of spermatogenic cells during the earlier stages of spermatogenesis. More likely, in the testis, they could be involved in spermatogenesis [43,44];
- MC4R, *Melanocortin 4 receptor*, is involved in different processes, such as energy expenditure, sexual activity and resistance to stressors [45];
- NPY, *Neuropeptide Y*, is probably the most studied peptide in the central feeding system [46]. It stimulate food intake in reptile as studied in red-sided garter snake, *Thamnophis sirtalis parietalis* [47];
- *Leptin*, the adipostatic hormone encoded by the *obese* (ob) gene, is generally secreted by adipocyte and provides feedback information to central receptors that control body weight homeostasis [48]. Some studies demonstrated that *leptin* is also expressed in another tissue like brain [49,50];
- PCNA, *Proliferating Cell Nuclear Antigen*, it plays an essential role in mitotic germinal epithelium [51].

To assay differential gene expression in two separate organs (testis and brain), the program RSEM (v1.2.30) [52] was used to map the trimmed reads (specific for individuals and tissues) against the mainland transcriptome assembly and to perform transcriptome expression quantification. Specifically, the following options were used for the analyses: “—bowtie2—bowtie2-sensitivity-level very_sensitive—forward-prob 0”. Effective counts were then used for a differential expression analysis with EBSeq (v 1.8.0) [53] using TMM (trimmed mean of M values) [54] normalization of the raw data. Gene Ontology Enrichment Analysis was performed on the list of differentially expressed transcripts by using the mainland annotation and in house-scripts. Specifically a hypergeometric test was performed to identify significantly enriched GO categories (FDR < = 0.05 after BH correction of the p-values).

The transcripts putatively involved in the MHC1, IgD, IgM, IgY, MC4R, NPY, *Leptin* and PCNA genes were identified by performing a BLASTn (v 2.2.30+) [55] search against the mainland transcriptome assembly. The results of the BLAST were filtered in order to retain those hits having an alignment length longer than 50% of the query and 30% of the hit (e-value < = 0.05).

## Results and discussion

The Italian wall lizard transcriptome presented here is a useful source for comparative genomics/transcriptomics, as well as for molecular ecology, developmental and evolutionary studies involving reptiles and island evolution. We successfully sequenced and assembled (with *de novo* method) the first large-scale, multi-organ transcriptome for *P*. *siculus*, developing a necessary resource for future reptilian evolutionary studies. A paired-end sequencing strategy was chosen to obtain short reads from the ends of longer DNA fragments in high-throughput sequencing (39,341,450.3 ± 6,094,476.7 Raw Data as number of reads, mean ± standard deviation). Quality control was performed on the raw sequencing data to remove low-quality portions while preserving the longest high quality portion of reads (% of Trimmed reads 93.9 ± 0.4, mean ± standard deviation). With high quality reads, after normalization, we performed an assembly for each sample separately (combining data from brain and testis of each sample) and then merged several assemblies for population (Table 1).

**Table 1:** Summary statistics for *de novo* assembly.

	Mainland	Island
N transcripts	275,310	269,885
%GC	45	45
Contig size range	250–18,833	250–16,779
Average Length	1,036.72	976.74
N50	1,704	1,576
Assembled bases (Mbp)	285	263

**Fig 2:**
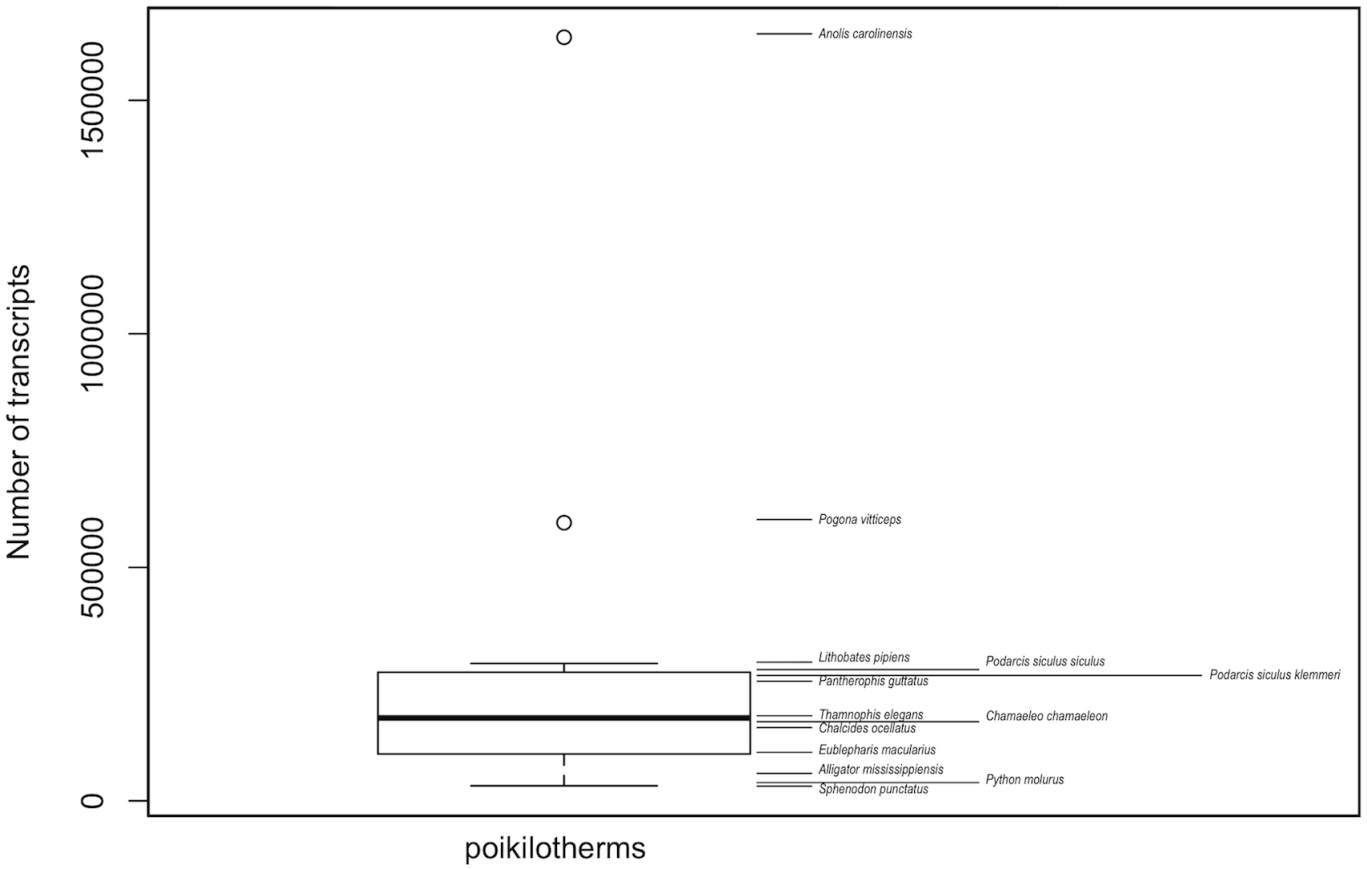
Number of transcripts contained in the transcriptomes of different poikilotherms. Number of transcripts for *P*. *s*. *klemmeri* (inhabiting Licosa island) and *P*. *s*. *siculus* (inhabiting the mainland, Punta Licosa).

The cleaned reads were deposited in the NCBI Sequence Read Archive (SRP074471). The number of transcripts in Italian wall lizard lied below the third quartile of the boxplot obtained using data from other reptiles (Fig 2) [56–58]. The number of transcripts with complete Open Reading Frame (ORF) and the corresponding proteins, using a minimum length of 50 aa for these latter, was 72,690 and 66,060 for mainland and island populations respectively (data in S1, S2 and S3 Appendices). In the BLAST search, results performed against the Sauropsida database proteins, showed 36,394 transcripts for the mainland population and 31,196 for the island population. Gene Ontology (GO) annotation was assigned to 18,434 and 21,606 proteins for the mainland and island populations, respectively, and multiple lines were used for a transcript in case of multiple annotations (data in S4 Appendix). Reads of each sample were mapped against the transcriptome of the mainland population. Percentage of mapped reads of our input RNA-seq reads were >80%, so they are more represented in our assembly, confirming the high quality of reference transcriptome (Table 2).

**Table 2:** Summary statistic from mapping reads of insular and mainland samples to the reference transcriptome.

Sample	Type	Total Mapped Reads	% Mapped
1	Mainland brain	21,968,821	87.25
2	Mainland brain	15,690,414	88.17
3	Mainland brain	15,087,168	84.71
4	Mainland testis	12,698,227	85.60
5	Mainland testis	17,028,041	88.36
6	Mainland testis	17,147,390	83.89
**Average**		**16,603,344**	**86.33**
7	Island brain	13,526,495	86.95
8	Island brain	15,171,423	86.45
9	Island brain	15,315,726	85.88
10	Island testis	17,107,734	84.17
11	Island testis	17,102,061	86.36
12	Island testis	12,766,982	83.43
**Average**		**15,165,070**	**85.54**

**Fig 3:**
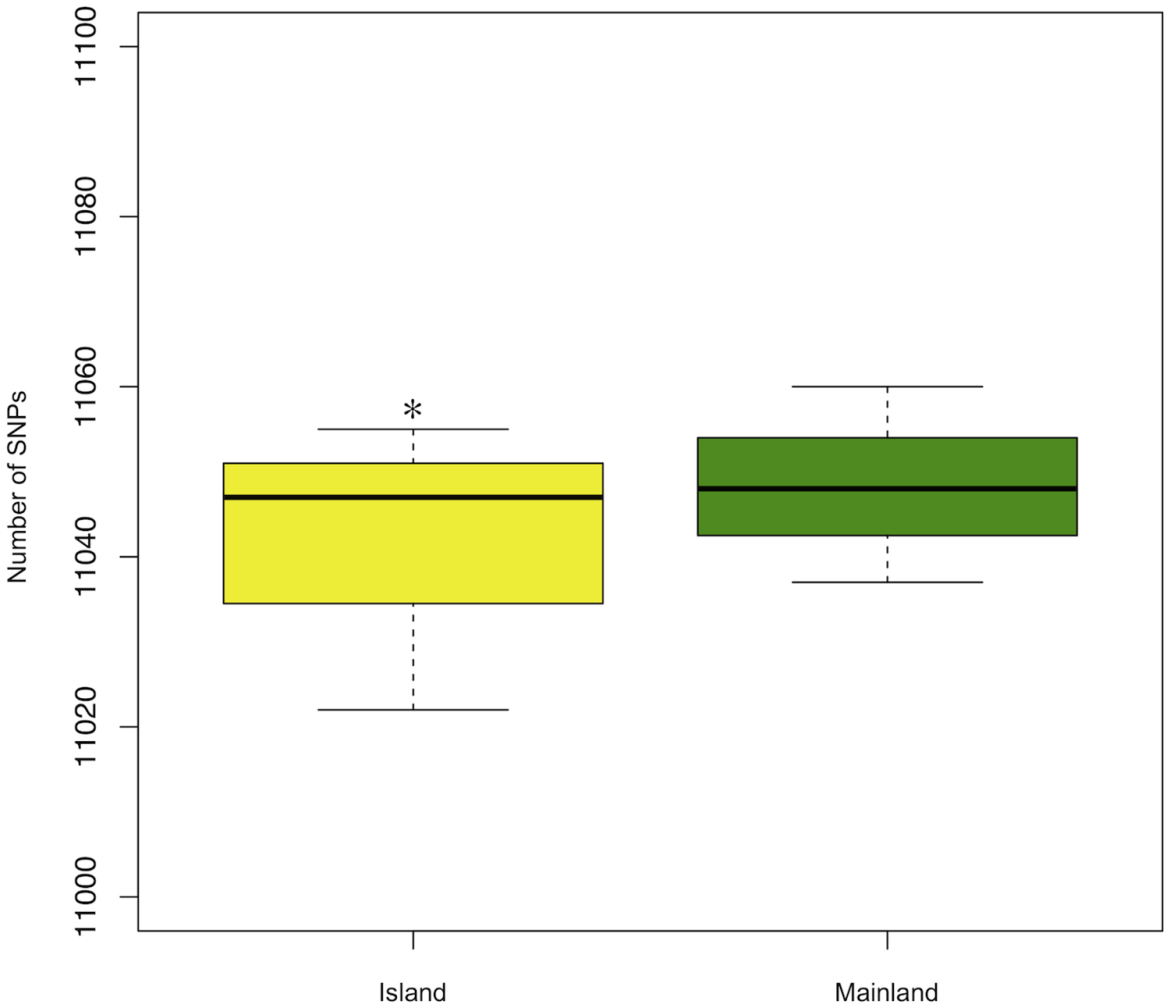
Number of SNPs in Island and Mainland samples. Number of polymorphisms (as mean ± standard deviation of number of SNPs) in common between two tissues in the two populations; t-test, *p>0.05.

From these mapped reads, only unique mapping reads were used to perform the variant calling analysis. We did not find a significant difference in the number of SNPs between individuals from mainland and island population (Fig 3). Probably the typical phenotypic traits of Syndrome are linked to differential gene expression rather than mutations in the coding regions. A total of 1,372 SNPs that were confirmed in the two tissues and in all sample of each population, were classified as being heterozygous (HZ) or homozygous (HM) (Table 3).

**Table 3:** Number of SNPs and relative transcripts in Island population against mainland reference transcriptome.

	SNPs	Transcripts affected by SNPs
Heterozygous	477	214
Homozygous	895	462

**Fig 4:**
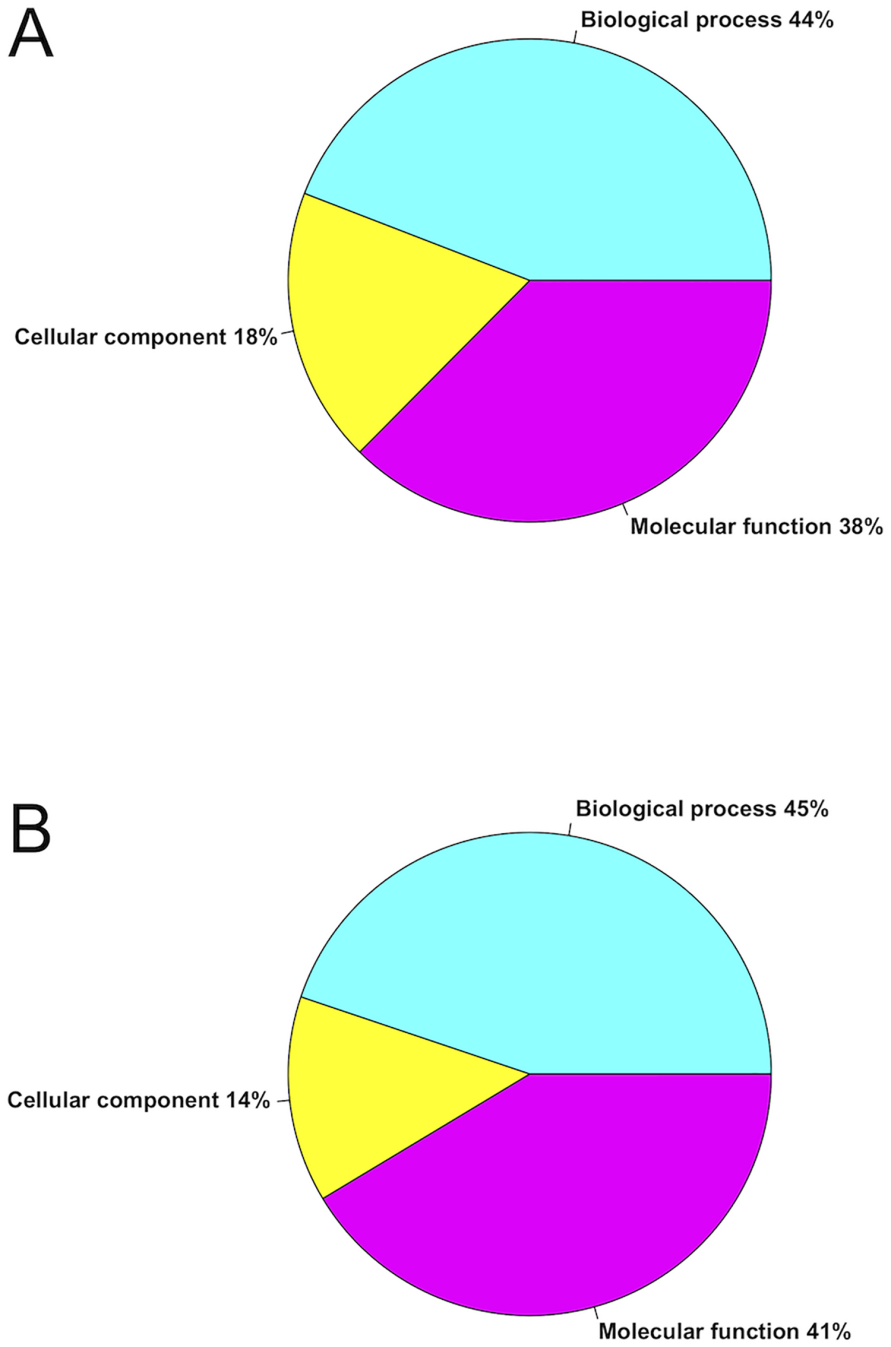
Gene Ontology Enrichment analysis of island population transcripts with SNPs. (A) SNPs in homozygous and (B) in heterozygous states.

**Fig 5:**
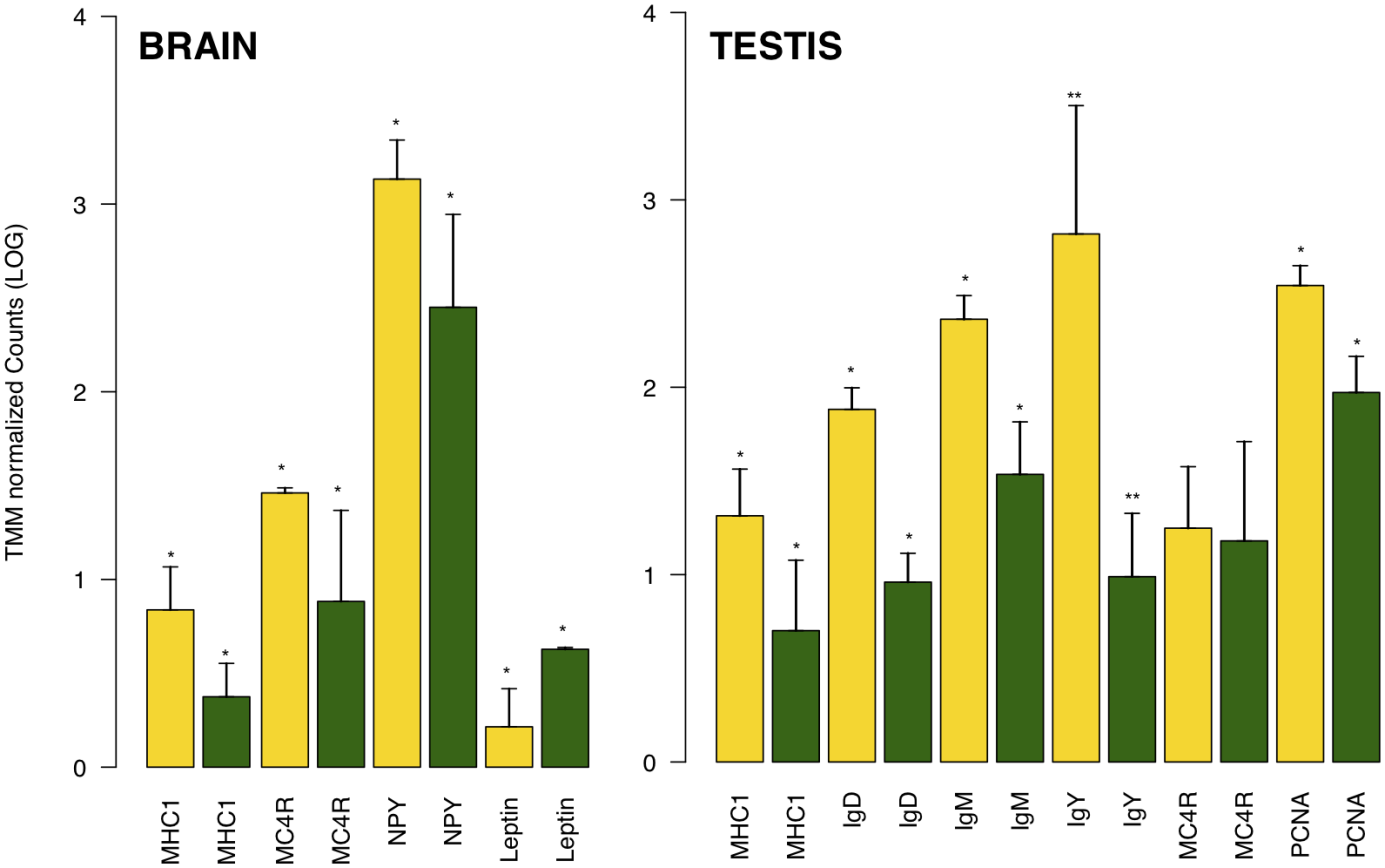
Differential gene expression in Island and mainland samples. Gene expression (as mean ± standard deviation of TMM Normalized Counts, LOG) in brain and testis of Island (yellow) and Mainland (green) samples; t-test, **p<0.01, *p<0.05.

Out of the 179 accession numbers of transcripts with SNPs in the HM state and enriched GO terms (FDR ≤ 0.05), 79 were annotated with a biological process, 67 with a molecular function and 33 with a cellular component (Fig 4A). Alternatively, out of 87 accession numbers of transcripts with SNPs in the HZ state and significantly enriched GO terms (FDR ≤ 0.05), 39 were annotated with a biological process, 36 with a molecular function and 12 with a cellular component (Fig 4B). The degree and type of variability within a contig can indicate selection in action. For the orthologous pairs with SNPs of the two populations, we determined whether the SNPs were non-synonymous polymorphisms (Ka) that changed the amino acid, or were synonymous polymorphisms (Ks). Out of 3,554 orthologous pairs 16 were with a Ka/Ks ratio >1 (p value ≤ 0.05, data in S5 Appendix). This indicates that mutations for these genes have changed the amino acid sequence more than would be expected under a neutral model, and that these genes may be under diversifying selection within or among the populations (or ecotypes) of the Italian wall lizard. The transcriptome assembly can provide a more complete picture of gene expression and structural variation and allows us to refine population genetic and evolutionary inferences. Differential gene expression represents a discriminative trait assayed either using Real Time-PCR on single genes (data in S6 Appendix, see also [15,20]), or using a transcriptomic approach (Fig 5). Through this latter method we found that in testis of insular lizards were more expressed genes like MHC1, involved in immune system defense, Igs, here involved in spermatogenesis [43,44] and probably linked to a rapid strong sexual maturation in insular lizards under syndrome, and PCNA, involved in nuclear cell proliferation. The brain of lizards from island also expressed MHC1 gene, but in addition it showed higher expression values for some genes such as MC4R and NPY, this latter involved in higher food intake attitude, a typical trait of lizards under syndrome [15] and as expected this was associated to lower values of *Leptin* gene expression. All of this was in accord with resistance to the stressors as well as intense sexual activity as described in syndrome [14,15]. Recent studies have identified signatures of positive selection or accelerated evolution in insular lizards driven by island syndrome that are related to development or functional structure. In our results, we support the critical role of regulatory changes in the evolution of insular lizard traits as a consequence of differential selection acting on island and mainland lizard populations. Although there are considerable differences between the island and mainland populations, probably Reversed Island Syndrome involves a suit of traits wider than considered genes. Future studies will be developed to complete the picture of the closely interested genes.

## Supporting information

S1 Appendix Transcripts with complete open reading frame. (TXT)

S2 Appendix Proteins sequences of transcripts from mainland population with complete open reading frame. (ZIP)

S3 Appendix Proteins sequences of transcripts from island population with complete open reading frame. (ZIP)

S4 Appendix GO annotation. GO annotation for all sequences.
(TXT)

S5 Appendix Orthologous transcripts pairs of island and mainland populations, investigated for Ka and Ks. (XLSX)

S6 Appendix Real Time-PCR. Method and results of Real Time-PCR conducted for some genes investigated through transcriptome analysis.
(DOCX)
