# Supplementary material for: The first transcriptome of Italian wall lizard, a new tool to infer about the Island Syndrome
Source: PLoS One. 2017 Sep 27;12(9):e0185227. doi: 10.1371/journal.pone.0185227 (PMC5617171; doi:10.1371/journal.pone.0185227)
Supplement: S6 Appendix — Method and results of Real Time-PCR conducted for some genes investigated through transcriptome analysis. (DOCX) [file pone.0185227.s006.docx]

S6 Appendix

Gene expression analysis by Real-Time PCR on MC1R, NPY and PCNA genes.

**Materials and Methods**

To determine the expression of MC1R, NPY and PCNA genes, total cellular RNA was isolated from tissues (skin (tail), brain and testis) of lizards from island and mainland population, by using TRI Reagent (EuroClone, Milan, Italy) according to manufacturer’s recommendations. The RNA quality and quantity were determined using Agilent Bioanalyzer 2100 (Agilent Technologies, Santa Clara, CA, USA) and Nanodrop spectrophotometer (Thermo Scientific Inc., Waltham, MA, USA) respectively. For cDNA synthesis with integrated removal of the genomic DNA contamination, QuantiTect® Reverse Transcription Kit (QIAGEN, Valencia, CA, USA) was used as described by the manufacturer. We conducted a RT-PCR by using SYBR® Premix Ex TaqTM II (Tli RNaseH Plus) RR820A (TaKaRa, Dalian, China). Quantitative RT-PCR analysis was conducted by using the 2(-ΔΔC(T)) method [[1](#_ENREF_1)]. RT-PCR was performed in a Rotor-Gene Q cycler (QIAGEN). Amplification curves, melting curves, and cycle threshold values (Ct values) were analyzed using Rotor-Gene Q Series Software (QIAGEN). For each RT-PCR experiment, data were normalized to the expression of the β-actin housekeeping gene.

The primers used for each gene were as follows: MC1R forward 5'-TGGAGACCCTCTTCATGCTTCT-3', reverse 5'-GCTGCAGATCAGCATGTCCA-3'; NPY forward 5'-CTCGGCGTTGAGACATTACA-3', reverse 5'-CACTTCCCATCACCACACAG-3'; PCNA forward 5'-AGTGAGAAGGCCTGGCAGTA-3', reverse 5'-CTTTGCAGCCAATCTCCTTC-3'; ACTB forward 5'-GATCTGGCACCACACCTTCT-3', reverse 5'-TCTTTTCTCTGTTGGCTTTGG- 3'.

Each sample was tested and run in duplicate and statistically significant expression changes were calculated using one-way ANOVA.

The mainland lizard was used to normalize the amount of mRNA in the insular population.

The results of this analysis were reported in Table 1. MC1R showed significant differences of expression, about 7-fold higher than the mainland wild-type, whereas NPY and PCNA genes exhibited a weak but significant over-expression in island population than mainland one (2-fold and 3-fold respectively).

| **mRNA fold** | | | |
| --- | --- | --- | --- |
|  | MC1R (skin) | NPY (brain) | PCNA (testis) |
| Island | 7 (±0.6) | 2(±0.3) | 3(±0.3) |
| Mainland | 1(±0.2) | 1(±0.2) | 1(±0.1) |
| ANOVA one way test | P<0.001; F=270; df=1 | P<0.01; F=23;  df=1 | P<0.001; F=120; df=1 |

**Table 1. Gene expression in the skin, brain and testis of lizards from island and mainland population. Results are expressed as mean (**± **standard deviation).**

We also evaluated the expression of the *Melanocortin-1 Receptor* (MC1R) gene in the skin, because it is associated with the strong melanization that characterizes these insular lizards [[2](#_ENREF_2)].

**References**

1. Livak KJ, Schmittgen TD. Analysis of Relative Gene Expression Data Using Real Time Quantitative PCR and the 2−ΔΔCT Method. Methods. 2001; 25: 402-408. <http://dx.doi.org/10.1006/meth.2001.1262>.

2. Fulgione D, Lega C, Trapanese M, Buglione M. Genetic factors implied in melanin-based coloration of the Italian wall lizard. Journal of Zoology. 2015; 296: 278-285. 10.1111/jzo.12242.
